# Comparison of the Properties of Pullulan-Based Active Edible Coatings Implemented for Improving Sliced Cheese Shelf Life

**DOI:** 10.3390/polym16020178

**Published:** 2024-01-07

**Authors:** Tamara Erceg, Olja Šovljanski, Ana Tomić, Milica Aćimović, Alena Stupar, Sebastian Baloš

**Affiliations:** 1 Faculty of Technology Novi Sad, University of Novi Sad, 21000 Novi Sad, Serbia; anav@uns.ac.rs; 2 Institute of Field and Vegetable Crops Novi Sad, National Institute of the Republic of Serbia, 21000 Novi Sad, Serbia; milica.acimovic@ifvcns.ns.ac.rs; 3 Institute of Food Technology Novi Sad, University of Novi Sad, 21000 Novi Sad, Serbia; alena.tomsik@fins.uns.ac.rs; 4 Faculty of Technical Science, University of Novi Sad, 21000 Novi Sad, Serbia; sebab@uns.ac.rs

**Keywords:** bilayer active edible coating, pullulan, chitosan, gelatin, hydrolate, synergistic effect of antimicrobials

## Abstract

The development of active edible coatings with improved mechanical and barrier properties is a huge challenge. In this study, active edible coatings for sliced cheese have been developed using pullulan (Pull) in combination with two different biopolymers, chitosan (CS) and gelatine (Gel), and a combination of hydrolats as a source of active compounds with antimicrobial effects. In comparison to the monolayer coating, the bilayer coating system demonstrates improved barrier and mechanical properties. A preliminary assessment of the antimicrobial effect of lemongrass and curry plant hydrolats has revealed that both hydrolats exhibited antimicrobial activity against the targeted bacterium *Staphylococcus aureus*, albeit at different levels. The obtained results suggest that a mixture of 1.56% lemongrass and 12.5% curry plant hydrolats yielded a lower fractional inhibitory concentration (FIC) value. Bilayer coating systems (Pull/CS and Pull/Gel) with an incorporated mixture of hydrolats have demonstrated effectiveness in both cases: artificial contamination before application of the coating system and after application of the coating system. In both contamination scenarios, the coating systems consistently effectively limited bacterial proliferation, indicating the antimicrobial effect of the hydrolat mixture in the coating layers. In the case of artificial contamination before applying the coating system, both coatings demonstrated antimicrobial effectiveness, but the formulation with chitosan had a biocide effect, while the other, with gelatine, had only a bacteriostatic effect in a long-term setting. In the second case, both Pull/CS and Pull/Gel coatings demonstrated effectiveness in inhibiting bacterial growth regardless of the moment of contamination of the sample; the Pull/CS coating showed slightly better antimicrobial activity, achieving complete elimination of bacteria earlier compared with the Pull/Gel coating system.

## 1. Introduction

In recent years, the demand for food packaging that addresses environmental concerns caused by petroleum-based plastic packages has increased, leading to the rise of natural biopolymer ingredients in food packaging applications. Switching from conventional synthetic polymers to biodegradable films should be emphasized as a highly desirable strategy, as well as developing sustainable active packaging materials that have a minimal impact on the health of consumers and the environment [1,2]. Such strivings have resulted in notable progress in the development and utilization of biopolymer-based edible coatings. These coatings, formed as continuous films or coverings for food products, contribute to extending shelf life, maintaining nutritional quality, and enhancing sensory attributes of foods [3,4,5]. Proteins, polysaccharides, and lipids represent an environmentally friendly alternative to synthetic polymers, offering potential solutions for reducing food waste and improving sustainability in the food industry [1,2,3,6,7,8,9,10,11]. A new generation of these coatings is designed to incorporate active ingredients such as colorants, flavors, enzymes, and antimicrobial compounds, enhancing the shelf life and nutritional value of packed products. The imperative to reduce weight loss and extend shelf life has driven innovation in biofriendly, sustainable, and inherently biodegradable packaging materials. Chitosan is one of the most important biopolymers used in the preparation of films and coatings, mostly due to its structural properties, barrier properties, and excellent antimicrobial and antioxidant characteristics [12,13]. However, its processing can be challenging, and chitosan is often modified or blended with other materials to improve its processability and tailor its performance to specific needs [8]. Recently, there has been notable advancements in the production of chitosan-based systems with multiple components and functions. Incorporating various bioactives, therapeutic agents, and conductive or magnetic components into these fibers is now a widely used technique to enhance and adjust the properties of chitosan and its derivatives [7,9,14]. Along with chitosan, polysaccharide pullulan and protein gelatine have been widely explored in edible coatings [15,16]. Pullulan, produced by the fungus *Aureobasidium pullulans* through fermentation, has great potential in the production of edible coatings [15] due to its superior film-forming and adhesive properties [16]. Gelatin, a highly abundant macromolecule obtained from fish and animal processing byproducts, is commonly used in edible coatings due to its ability to form a flexible and transparent film. It is a regularly used edible component for food packaging because of its good barrier capacity, transparency, and high contents of proline, glycine, and hydroxyproline, which provide the gelatine with exceptional film-forming ability, thus enabling the formation of a flexible film [8]. For a long time, biopolymers were applied mostly as a one-component film or coating formulation [7]. Recent advances in edible films involve diverse biopolymers utilized in polymer blend preparation and two-layered or multi-layered film formation, with or without added active compounds. The reasons for these strategies lie in obtaining better film-forming ability, barrier properties, and functionality of edible films, but also the possibility for more easily adding active compounds. Chitosan, gelatin, and pullulan have been investigated in combination with each other, and with starch, whey, carboxymethyl cellulose, and alginate, using the design of polymer blends in food packaging [17,18,19,20,21,22,23]. Combining chitosan, pullulan, and gelatin, among others, in two-layered or multi-layered edible films enhances barrier, mechanical, and functional properties for tailored food packaging and preservation [24,25]. As a cationic biopolymer able to form hydrogen and ionic interactions on the contact surface with other hydrophilic biopolymers, chitosan is highly investigated [26]. In addition, modifying the film structure through cross-linking reactions can enhance the cohesion between polymer chains and active compounds, resulting in better retention within the polymer network and improved control over their release [27]. The multi-layer coating based on pullulan and chitosan applied in two, four, and six layers on the fresh papayas investigated by Zhang et al. has been presented as a versatile strategy for meeting specific requirements in food packaging and preservation [28]. The combination of chitosan, pullulan, and mucilages has shown promise in preserving freshly cut pineapple, while multi-layer coatings based on pullulan and chitosan have been investigated in preserving fresh pineapple [29]. Different essential oils with strong antimicrobial potential (clove essential oil, lemongrass essential oil, etc.) have been used as active compounds in these formulations. However, the incorporation of essential oils in a hydrophilic biopolymer matrix requires the use of surfactants and different methods of encapsulation, considering the difference between oil and biopolymer polarity. Encapsulation makes the whole procedure more complicated, expensive, and time-consuming. Also, some essential oils have strong flavors and odors that might not be desirable in certain food products or could overpower the original taste [30]. Using hydrolats as a source of antimicrobial compounds in edible coatings is a quite new approach that is important from ecological and economic aspects for the implementation of a bio-based circular strategy in the food and pharmaceutical industries. Hydrolats (hydrosols) are byproducts of the steam distillation process used to extract essential oils from plants. They contain water-soluble compounds and trace amounts of essential oils [31]. Hydrolats do not possess the same level of antimicrobial efficacy as essential oils due to their lower concentration of active compounds. However, they are compatible or miscible with hydrophilic biopolymers such as pullulan, chitosan, and gelatin. On the other hand, efficient antimicrobial activity can be achieved using a greater amount of hydrolats during the preparation of edible coating formulations. Greater economic production and incorporation in the biopolymer matrix align with the industry’s pursuit of sustainable alternatives to synthetic packaging, addressing both the need for improved food preservation and the demand for environmentally friendly solutions. Furthermore, the integration of plant hydrolats offers an additional dimension of bioactivity, where the antimicrobial and antioxidant compounds from hydrolats work in tandem with the film structure to inhibit bacterial growth and enhance overall food preservation. This has been confirmed by the results of the investigation conducted by Didar, who coated eggs with a combination of pectin and cinnamon hydrolat, providing improved structural uniformity and enhanced microbial quality throughout a six-week storage period [32].

In cheese production, microbiological contamination encompasses a spectrum of microorganisms, prominently including bacteria and fungi. The surface of cheeses is liable to contamination by microorganisms because of favorable acidity and high water activity. The severity of the problem is reflected in the fact that the majority of the research conducted on cheese storage and shelf life is on the prevention of and reduction in microbial contamination [33]. Furthermore, the loss of significant amounts of moisture in some types of cheese due to poor barrier properties of the packaging or no packaging at all results in the formation of undesired sensory properties. *Staphylococcus*, notably *Staphylococcus aureus*, emerges as a concern due to its capacity to produce heat-stable enterotoxins, posing potential health risks if not adequately addressed. The prevention of *Staphylococcus* contamination in cheese hinges on rigorous hygiene practices during milk handling and processing, with regular testing being crucial to ensure product safety [34]. Stringent hygiene measures are essential for controlling *Staphylococcus*, emphasizing personnel, equipment, and environmental cleanliness, but required control measurements (maintaining proper storage conditions, minimizing cross-contamination, and adhering to good manufacturing practices) are often absent in real conditions. Regular testing for *Staphylococcus* and monitoring for visible signs of fungal growth are imperative elements of quality assurance in cheese production. The multifaceted nature of these contaminants necessitates a comprehensive approach to ensure both safety and quality throughout the cheese-making process [35]. The potential of developing bio-based coating systems for foods, with a focus on inhibiting the growth of *Staphylococcus aureus*, is promising. Some chitosan-based composites with *Eucalyptus globulus* essential oil have the capacity to form protective barriers, preventing the proliferation of *Staphylococcus aureus* on food surfaces [36]. A similar effect was observed in the case of the addition of tea extracts or nisin in chitosan bases [37,38,39]. 

A combination of pullulan/chitosan and pullulan/gelatine in the design of active bilayer coatings intended for the preservation of cheese, using hydrolats as a source of active compounds, has not been investigated according to the available literature data. 

Therefore, the aim of this study was to formulate pullulan/chitosan and pullulan/gelatine two-layered edible films with enhanced preservation properties by incorporating active compounds of curry plant and lemongrass hydrolats for the extension of the shelf life of the sliced cheese and to compare their barrier and mechanical properties, as well as antimicrobial potential. These biopolymers, as well as plasticizer and hydrolats, are considered edible substances and have been extensively studied and used in various edible applications, including food additives, encapsulation, viscosity correctors, and the preparation of edible coatings [16,17,18,19,20,21,22]. Chitosan and gelatine are widely used in the food industry, and they are generally regarded as safe for consumption by regulatory bodies such as the U.S. Food and Drug Administration (FDA). Pullulan is used as a food additive. It is commonly used in food packaging, and it is generally recognized as safe (GRAS) by the FDA. Glycerol is a sugar alcohol commonly used in processed food, beverages, and candies as a sweetener, solvent, and humectant. It is recognized as safe for consumption by regulatory authorities such as the U.S. Food and Drug Administration (FDA) and the European Food Safety Authority (EFSA). Lemongrass is a culinary herb commonly used in various Asian cuisines for its citrusy flavor. The leaves of curry plant hydrolat have been commonly used to make herbal teas. 

Since the prevalence of *Staphylococcus aureus* in the cheese industry poses food safety risks, with contamination occurring at various production stages, effective coating systems are vital for preventing bacterial entry and ensuring product quality. Therefore, this study is aimed at making progress in coating systems for food application by comparing the properties of pullulan—based active edible coatings. Additionally, their implementation for improving cheese shelf life was demonstrated through in vitro testing on sliced cheese samples.

## 2. Materials and Methods

### 2.1. Materials

Pullulan (Mw~574,570 g/mol) was supplied from Avena Lab (Vršac, Srbija). Chitosan (Mw = 100,000–300,000 g/mol) was purchased from Sigma Aldrich (St. Louis, MO, USA), while glacial acetic acid, gelatine, and glycerol were purchased from Centrohem (Stara Pazova, Serbia). The preparation of the film-forming solution was carried out in a mixture of lemongrass—*C. citratus (DC) Staph* and curry plant—*H. italicum (Roth) G. Don* hydrolats. *Staphylococcus aureus* ATCC 25923 was obtained from the American Type Culture Collection (ATCC), Manassas, VA, USA. All chemicals and nutrient media for microbiological testing were purchased from HiMedia (Mumbai, India). 

The analysis of volatile compounds presented in hydrolat was performed through simultaneous steam distillation and extraction using the Likens–Nickerson apparatus (with the addition of CH_2_Cl_2_) for 2 h. Further, gas chromatography with a flame ionization detector (GC-FID) and gas chromatography coupled to mass spectrometry (GC-MS) using an Agilent 7890A GC (Santa Clara, CA, USA) were performed. The main compounds in the lemongrass hydrolat were geranial (32.6%) and neral (26.1%), while in the curry plant hydrolat, the main compounds were α-terpineol (14.3%) and linalool (14.2%). Chromatograms are given in the Supporting Material (Figures S1 and S2). 

### 2.2. Preparation of Hydrolates

A small-scale distillation unit at the Institute of Field and Vegetable Crops Novi Sad was used for the steam distillation of lemongrass (*Cymbopogon citratus*) and curry plant (*Helichrysum italicum*). The dried plant material (about 100 kg) was placed in a distillation vessel made of stainless steel (volume 800 L). Steam was generated by a high-pressure boiler, and a piping system supplied the distillation vessel with plant material from the bottom. The volatile compounds from the plant material, together with water vapor leaving the stainless steel vessel via the top pipe, entered a water-cooled condenser and cooler, and then moved further into a glass Florentine flask, where water condensate (hydrolat) was separated from the essential oil (floating on top). 

### 2.3. Preparation of Film-Forming Solutions for Edible Coatings

Pullulan (10 wt%) was added to the mixture of lemongrass hydrolat and curry plant hydrolat and stirred at 55 °C until complete dissolution. A chitosan solution (5 wt%) was prepared in the mixture of hydrolats with glacial acetic acid that was added to adjust pH to 4.5 and was stirred until complete dissolution at 55 °C. The gelatine solution (5 wt%) was prepared in a similar way as the pullulan and chitosan solution but with the addition of gelatine to the mixture of lemongrass hydrolat and curry plant hydrolat, followed by stirring at 55 °C until it had completely dissolved. The biopolymers were dissolved in the mixture which contained 1.56% lemongrass and 12.5% curry plant hydrolats, while the biopolymer/hydrolat weight ratio was as follows:

Pull/lemongrass hydrolat = 1:1.56; Pull/curry plant hydrolat = 1:12.5.

CS/lemongrass hydrolat = 1:3.12; CS/curry plant hydrolat = 1:25.

Gel/lemongrass hydrolat = 1:3.12; Gel/curry plant hydrolat = 1:25.

The prepared solutions were poured into Petri dishes and dried at 45 °C for 5 h. Circle-shaped samples were used for the determination of barrier properties and the preparation of samples for mechanical analysis.

### 2.4. Characterization Techniques

The chemical structure of bilayer films was analyzed by Fourier-transform infrared spectroscopy (FTIR) (Waltham, MA, USA) operating in total reflectance attenuation (ATR) mode. All spectra were captured with a resolution of 4 cm^−1^ in the spectral range of 4000–400 cm^−1^.

Using a scanning electron microscope (JEOL JSM-6460, Tokyo, Japan) with an accelerating voltage of 20 kV, the microstructure of polymer blends was examined. Samples were cut, gold sputter-coated, and examined using the SCD-005 (Bal-tec/Leica, Wetzlar, Germany) apparatus after being submerged in liquid nitrogen.

Moisture content was determined using a Halogen HE53 Moisture Analyzer–METTLER TOLEDO (Petaling Jaya, Malaysia). Rectangular-shaped samples (approximately 0.3 g) were used for the determination of moisture content. Measurements were conducted in triplicate at a temperature of 105 ± 1 °C. 

The thickness of the films was measured using a micrometer DML DC04150 Digital Caliper, with an accuracy of ±0.02 mm (Sheffield, UK), at 9 positions, using three samples, and the average value was used.

The Instron Universal Testing Instrument Model 1122 (Instron Engineering Corp., Canton, MA, USA) was used to examine the tensile properties of bilayer films. The samples were prepared and tested according to the ASTM standard D882-18 [40]. The samples were cut into rectangular shapes (80 × 15 mm). All tests were performed at a temperature of 23 ± 2 °C with an initial grip spacing set to 50 mm and a crosshead speed of 10 mm/min. The results were averaged from three independent measurements and extreme values were excluded.

The water vapor permeability (WVP) of bilayer films was determined gravimetrically using a modified ASTM Method E 96, at a relative humidity of 75% and temperature of 25 ± 1 °C (ASTM 96, 2017) [41] during the 6 h. Steel cups filled with distilled water were sealed with films, weighed, and placed in a desiccator with silica gel. While the relative humidity within the desiccator was set at 0%, it was fixed at 100% inside the cups. Equation (1) was used to determine the WVP (g/h·m·Pa):

(1) $$WVP\;(g/h\cdot m\cdot Pa)=\frac{w}{t\cdot A}\cdot \frac{L}{∆p}$$

where A is the film’s exposed area (0.002375 m^2^), L is the film’s thickness (m), and Δp is the partial water vapor saturation pressure across the film (3169.22 Pa). W/t is the slope of the straight line expressed in grams per hour. For every sample, the tests were carried out in triplicate.

The thermal decomposition of bilayer films was investigated by the LECO 701 Thermogravimetric Analyzer (St. Joseph, MI, USA). The samples (about 0.3 g) were heated from room temperature to 800 °C nitrogen atmosphere at a heating rate of 10 °C/min and a flow rate of 50 mL/min.

The bacterial growth inhibition effect of individual hydrolats was evaluated by the disk diffusion method described in detail by Aćimović et al. [42]. This test was conducted against *Staphylococcus aureus* ATCC 25923, a bacterium commonly associated with cheese contamination during production and storage processes [43]. The methodology involves utilizing a freshly prepared suspension (~6 log CFU/mL, prepared using McFarland standards) of overnight bacteria culture to inoculate a suitable nutrient Miller-Hinton medium. Subsequently, 15 μL of the tested hydrolats is applied to three sterile cellulose discs previously placed on the solidified medium. After the incubation period (37 °C for 48 h in aerobic conditions), halo zones around the discs are measured. The interpretation of antimicrobial potential results is based on the size of inhibition zones: resistant (zone < 22 mm), intermediary effect (zone between 22 and 26 mm), and sensitive (zone > 26 mm).

The synergistic effect of two hydrolats was tested using the checkerboard assay as a well-established method for the determination of synergistic or antagonistic relationships between samples [44]. This assessment is primarily based on the determination of minimal inhibitory concentrations of individual hydrolats (MIC_A_ and MIC_B_). After the formation scheme of a series of two-fold dilutions for each hydrolat across (initial concentration was defined as 100%, as the following concentrations were 50, 25, 12.5, etc.) the rows and columns of the microtiter plate, a fixed concentration of bacterial cells was added. Subsequently, the varying concentrations of hydrolats were added to each well following the formed scheme on the microtiter plate. Control wells with the targeted bacterium were included as well as each hydrolat individually at different concentrations. The same nutrient medium and incubation conditions as for the previous step were used for the microtiter plate incubation. After the incubation period, an examination of the wells for visible growth was performed, and the fractional inhibitory concentration (FIC) for each combination of hydrolats (Equation (2)) was calculated. In this case, the FIC represents the sum of the fractional inhibitory concentration of the lemongrass hydrolat (FIC_A_) and the fractional inhibitory concentration of the curry plant hydrolat (FIC_B_).

(2) $$FIC={FIC}_{A}+{FIC}_{B}=\frac{{MIC}_{A\;in\;combination}}{{MIC}_{A\;individually}}+\frac{{MIC}_{B\;in\;combination}}{{MIC}_{B\;individually}}$$

If the FIC is less than or equal to 0.5, the synergistic effect can be summarized, meaning that the combined action of hydrolats is more effective than their individual antimicrobial effects. The result between 0.5 and 1 suggests an additive effect, where the combined action is simply the sum of the individual effects. Lastly, an FIC value greater than 1 shows an antagonistic effect, where the combined action is less effective than the individual effects.

Determined MIC values of both hydrolats, as well as hydrolat concentration in the synergistic combination, were served for further testing of accessibility in an active packaging system. The bilayer formations of the developed packaging system were tested using a similar methodology as described in the previous step. This step was conducted for coatings formed without hydrolats (as controls) and with hydrolats (as active systems). For each layer in its liquid form, 15 μL aliquots were applied onto sterile cellulose disks (n = 3). The incubation and determination of inhibition zones for both hydrolats were conducted following the same incubation conditions as for the previous step. The examination encompassed packaging systems without the incorporation of essential oils or hydrolats (controls), as well as active systems containing these components.

### 2.5. Implementation of Edible Coatings as an Antimicrobial Active System

For the antimicrobial assessment, cheddar-type cheese slices were utilized as test samples. Each sample had an average mass of 10 g and a thickness of 1.5 mm. The cheese slices were procured from a local dairy manufacturer situated near Novi Sad, Serbia, on the initial day of production. Following collection, the samples were transported under cold chain conditions to the Laboratory for Microbiology at the Faculty of Technology in Novi Sad. The recommended shelf life for the cheese slices is 14 days when stored under refrigeration (4–10 °C). The cheese slices underwent a series of preparatory steps before the application of the edible coatings to ensure the elimination of initial contamination. UV light exposure was carried out on both sides of the slices following a method described by Lee, Lee, and Song [45], while the complete treatment (intensity of 3 mW/cm^2^, 15 min) was conducted in a UV cabinet with 4 UV-C lamps which were positioned at 2 cm from the cheese sample surfaces. 

Afterward, the samples were divided into two parts for subsequent testing. In the first part, artificial contamination with *S. aureus* was introduced before the application of the packaging system to mimic primary production contamination. The contamination was achieved by inoculating the slices with an *S. aureus* suspension containing sensitive bacterium at a concentration of approximately 7 log CFU/mL. Each slice was inoculated with 100 μL of the suspension (50 μL on each side), resulting in a final *S. aureus* concentration of approximately 5 log CFU/slice (corresponding to 4 log CFU/g of cheese sample). In the second part, artificial contamination was introduced after the application of both packaging layers to simulate contamination during the packaging and distribution processes. All tests were conducted in triplicate, and the results are presented through kinetic pathways with error bars which implicate the calculated standard deviation in MS Excel program.

**Figure 1:**
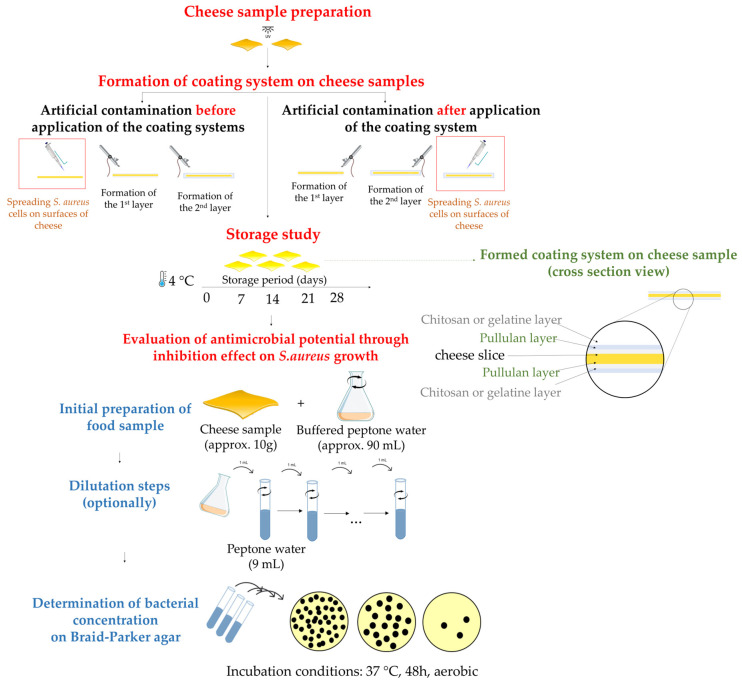
Illustration of coating systems formation on cheese samples and antimicrobial assessment.

A simplified scheme of the preparation steps as well as the following steps in antimicrobial assessment are presented in Figure 1.

**Figure 2:**
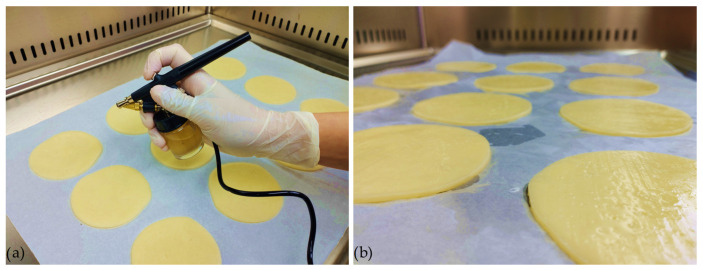
Application of coating system on cheese samples: (**a**) spraying with a pneumatic sprayer; (**b**) freshly applied coatings on samples.

To optimize the adherence of the coatings, the bilayer of the packaging system was applied using a spraying method with a small-scale pneumatic sprayer (Figure 2). The sprayer employed a suction system with a nozzle caliber of 0.3 mm, and the working pressure ranged from 1 to 3.5 bar. The control of liquid and air dosing was performed simultaneously in one step. The thickness of the samples was in the range between 0.33 and 0.38 mm, determined after drying on the cheese surface and peeling of the films. 

After preparation, the system was stored at 10 °C in a temperature-controlled incubator for 28 days. Sampling and analysis were conducted at regular intervals (0, 1, 3, 7, 14, 21, and 28 days). For each sampling time, the whole slice samples were mixed with peptone water in a 1:10 ratio using an EasyMIX blender (BioMerieux, Marcy-l’Étoile, France). Subsequently, a series of dilutions were prepared, and aliquots were streaked onto selective media for each sensitive bacterium (Baird–Parker agar). After incubation, colonies were counted, and the results were expressed as log CFU/g.

## 3. Results and Discussion

### 3.1. Fourier-Transform Infrared Spectroscopy (FTIR)

**Figure 3:**
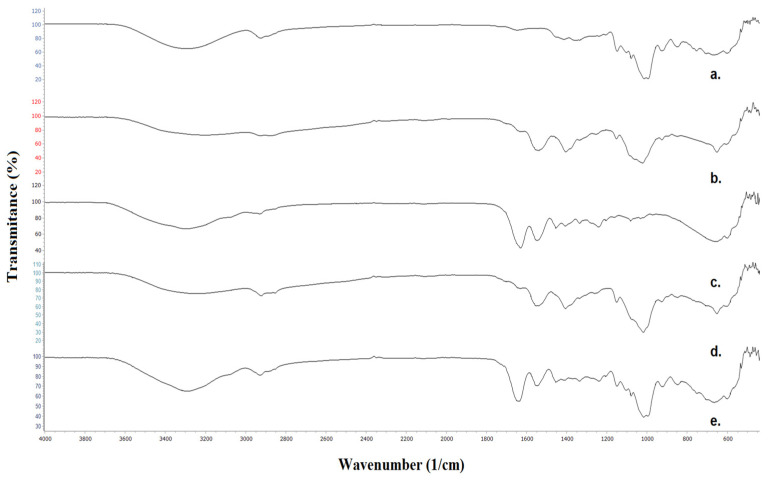
FTIR spectra of (a) Pull film, (b) CS film, (c) Gel film, (d) Pull/CS bilayer film with incorporated hydrolats, and (e) Pull/Gel bilayer film with incorporated hydrolats.

As shown in Figure 3, a broad peak with the center at 3287 cm^−1^ in the FTIR spectrum of neat Pull corresponds to the OH stretching originating from the hydroxyl groups. A broad peak with the canter at 3195 cm^−1^ is attributed to the N-H stretching and OH stretching. In the spectrum of the bilayer film, the center is shifted at 2252 cm^−1^. Two bands due to CH stretching appear at 2925 and 2887 cm^−1^ in the FTIR spectrum of Pull and 2927 and 2876 cm^−1^ in the spectrum of CS. In the spectrum of the bilayer film, in this range, three bands are attributed to CH stretching (at 2921, 2881, and 2852 cm^−1^), originating from CH_2_ groups in Pull, CS, and CH_2_ and aldehyde groups in hydrolats. 

The signal at 1645 and 1636 cm^−1^ in the spectrum of Pull, CS, and the bilayer film is attributed to the stretching of the O-C-O bond in the polysaccharide skeleton. N-H bending in the secondary amide appears as a peak at 1540 cm^−1^ in the spectrum of CS and the bilayer film. Two peaks between 1410 and 1335 cm^−1^ in all spectra are attributed to CH_2_, and CH_3_ bending. The peak at 1256–1240 cm^−1^ in all spectra corresponds to the vibration of the OH group, while the peak between 1154 and 1150 cm^−1^ is attributed to the C-O-C stretching of the glycosidic bond. 

The absorption bands in the range 1080–1015 cm^−1^ correspond to C-C-O stretching and C-O-H bending. A small peak at 926 cm^−1^ is attributed to the glycosidic bond in polysaccharides. Similar absorption bands to that of CS were noticed in the FTIR spectrum of Gel. The overlapping of N-H and OH stretching vibrations of amino acids in gelatine results in a broad absorption band between 3600 and 3000 cm^−1^, with a peak at 3292 cm^−1^. The presence of an aromatic ring originating from a gelatine–amino acid constitutive unit is confirmed by a weak peak at 3073 cm^−1^, visible in the spectra of a neat Gel and Pull/Gel bilayer film. Three peaks between 2927 and 2854 cm^−1^ are visible in the FTIR spectrum of the bilayer film with incorporated glycerol and hydrolats. An intense peak at 1644 corresponds to CO stretching vibrations, while a peak at 1549 (Gel) and 1544 cm^−1^ (Pul/Gel) is attributed to the NH bending and C-N stretching. The stretching of the COO- group appears as a weak peak at 1454 cm^−1^. 

### 3.2. Scanning Electron Microscopy (SEM) Analysis

**Figure 4:**
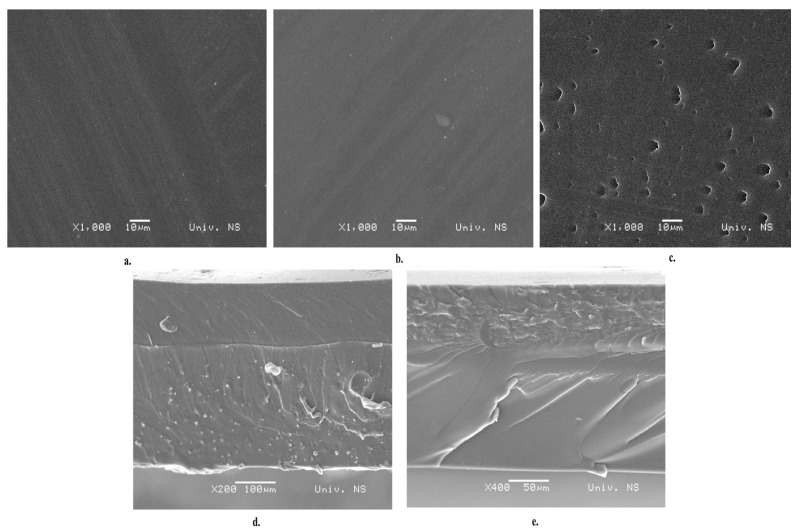
SEM images of (**a**) Pull film, (**b**) CS film, (**c**) Gel film, (**d**) cross-section of Pull/CS film, and (**e**) cross-section of Pull/Gel film.

SEM micrographs of the monolayer film surface and bilayer film cross-sections are shown in Figure 4. Cross-sections of the bilayer films (Figure 4d,e) reveal the existence of good adhesion between Pull and CS, as well as between the Pull and Gel layers. Good adhesion is the result of hydrogen bonding, and it is desirable as it is the starting point for achieving better barrier and mechanical properties. 

### 3.3. Mechanical Property Analysis

The results of mechanical characterization—tensile strength (TS) and elongation at break (EB)—for the monolayer and bilayer films are summarized in Table 1. Due to the formation of secondary bonding on the Pull/CS and Pull/Gel surface, the TS of the bilayer films is up to four to nine times greater than for the monolayer films, also due to the hydrogen bonds formed at the interface between the two layers that provide additional adhesion which contributes to the film’s overall tensile strength. It is a significant improvement in comparison to the results obtained by Ferreira et al., for FucoPol/CS bilayer films, and Wang et al., for Kafirin/Gel multi-layer films, where the obtained TS values were lower than for the CS monolayer film [46,47]. It can be observed that the Gel monolayer film possesses a greater value of TS in comparison to the Pull and CS monolayer films, probably due to the presence of hydrogen bonds and ionic interaction between the macromolecular chains, because Gel as polyampholyte possesses positive and negative charged groups (amino and carboxylic). Improved mechanical strength provides structural support to the cheese, reducing the risk of physical damage during handling, transportation, and storage. Also, they correlate to the covering ability and barrier properties of the coatings.

### 3.4. Barrier Properties—Water Vapor Permeability (WVP) Measurements

The values of WVP of the monolayer and bilayer films, along with thickness and moisture content, are summarized in Table 2. Neat pullulan and chitosan films (monolayer) have shown the highest WVP values (1.4 × 10^−3^ and 1.3 × 10^−3^ g/m^2^·h·Pa). Bilayer films have been shown to significantly lower values of WVP in comparison to neat monolayer films, which confirms the hypothesis about improving barrier properties using the design of bilayer films. In comparison to the multi-layer FucoPol/CS and Kafirin/gelatine films, the barrier properties of bilayer films are significantly improved, as well as in comparison to the monolayer Pull, CS, and Gel films [46,47]. Enhanced barrier properties can regulate moisture exchange between the cheese and its environment. This helps prevent excessive moisture loss or uptake, acting at the same time as a shield against microbial contamination and thus reducing the risk of mold growth or bacterial spoilage on the cheese surface.

### 3.5. Thermogravimetric (TGA) Analysis 

**Figure 5:**
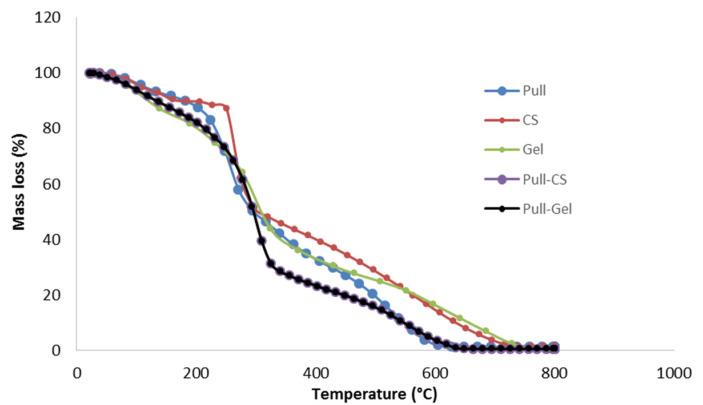
TGA thermograms of monolayer (Pull; CS) and bilayer films (Pull/CS; Pull/Gel).

The prepared films’ TGA curves are displayed in Figure 5. Table 3 summarizes the values of maxima degradation rates obtained from the derivative thermogravimetric curves (DTG). Thermal decomposition of the films is carried out in three main stages: the first stage, up to 158–184 °C, with weight loss of up to 9%, represents the loss of free and bound water absorbed in the film. The second and main degradation stage (up to 320–357 °C) with a weight loss of up to 74% corresponds to the breaking of glycoside linkages in Pull and CS and amide linkages in Gel, the decarboxylation of Gel, and glycerol degradation. The weight loss in the third stage is attributed to the complete decomposition of the biopolymer backbone and continues up to 0.02% of the residual weight.

### 3.6. Antimicrobial Potential of Plant Hydrolats, Control, and Active Coating Layers

The individual antimicrobial effects of the two tested hydrolats, lemongrass and curry plant, against the targeted bacterium *Staphylococcus aureus* were investigated and are presented in Table 4. The results reveal that both hydrolats exhibited antimicrobial activity, albeit at different levels. The lemongrass hydrolat exhibited a significant inhibition zone of approximately 34.33 mm. This indicates that the lemongrass hydrolat has a potent antimicrobial effect against the target bacterium. The large inhibition zone suggests that the lemongrass hydrolat is highly effective in inhibiting the growth and proliferation of the target bacterium. Such a substantial zone of inhibition indicates a broad range of antimicrobial action and could make the lemongrass hydrolat a promising candidate for further investigation as a natural antimicrobial agent. The curry plant hydrolat displayed an inhibition zone of approximately 25.0 mm. This indicates a considerable antimicrobial effect against the target bacterium, though it is slightly smaller compared to the lemongrass hydrolat. However, an inhibition zone of 25.0 mm still signifies substantial antimicrobial activity, and the curry plant hydrolat may have a valuable role in inhibiting bacterial growth in certain applications. 

**Figure 6:**
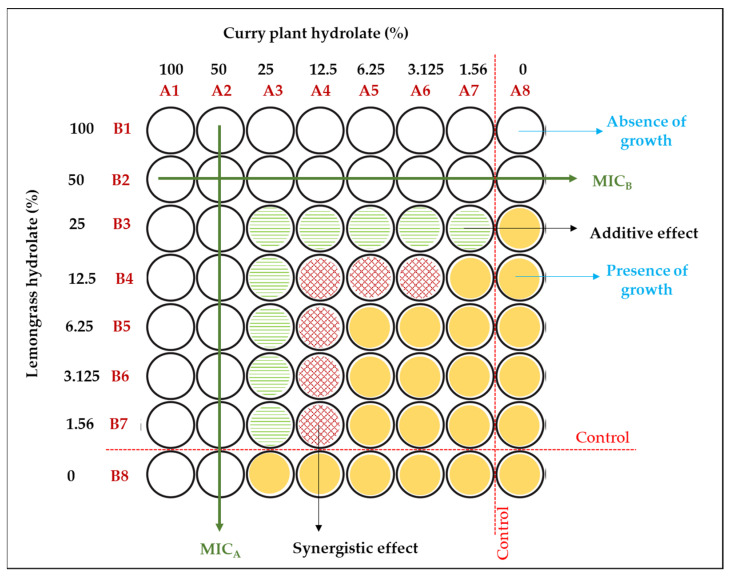
Visual presentation of checkerboard testing of the synergistic effect of lemongrass and curry plant hydrolats against *Staphylococcus aureus*.

The observed differences in antimicrobial efficacy raise the possibility of a potential synergistic effect between the two hydrolats. Therefore, it was essential to consider that the results presented are based on inhibition zone assays that have to be fulfilled with an investigation on the potential synergistic effect of these two hydrolats. To explore this further, a checkerboard assay was conducted as part of this study through a checkerboard test (Figure 6). 

In Figure 6, a visual representation of the mutual use of two tested hydrolats against the targeted bacterium is presented. The results indicate that the minimum inhibitory concentrations (MICs) of both hydrolats were half of their initial values (considering the initial concentration of the crude hydrolat as 100%). The obtained results reveal a noteworthy reduction in the minimum inhibitory concentrations (MICs) of both hydrolats to half of their initial values (marked as 100% since crude hydrolat was used). Namely, a synergistic effect calculated as the fractional inhibitory concentration (FIC) between the hydrolats was observed at significantly lower concentrations, specifically ranging from 12.5% to 1.56% for the lemongrass hydrolat in combination with the curry plant hydrolat, with both hydrolats falling within the same concentration variation range (a calculated fractional inhibitory concentration (FIC) value lower than the limitation of 0.5 indicates a synergistic effect of the tested substances). Concentration combinations that fell between the absence of growth and the synergistic effect were found to indicate an indifference effect. These particular combinations exhibited either no increase in inhibitory activity or only a slight increase, resulting from the additive effect of the combined hydrolats [45]. The observation of a synergistic effect at these lower concentrations of individual hydrolats indicates that the combined hydrolats exert a stronger antimicrobial action compared to their individual use. An in-depth analysis of different concentration combinations of lemongrass and curry plant hydrolats against bacterial growth was undertaken. Notably, the lowest FIC value was observed at a specific combination of 1.56% lemongrass hydrolat and 12.5% curry plant hydrolat, signifying the most significant synergistic effect at these concentrations. Summarily, these results provide valuable research insights into the synergistic antimicrobial effect achieved through the combination of plant hydrolats. The identification of lower MICs and the pronounced synergistic effect at specific concentrations emphasize the potential of these hydrolats as promising natural antimicrobials in specific systems for food application. Therefore, optimizing antimicrobial synergy in the context of combined hydrolat treatments offers opportunities for more effective and efficient approaches to control bacterial growth in coating system applications.

After bilayer formation, the in vitro antimicrobial potential of both coating layers with and without added hydrolats was also evaluated in view of antimicrobial potential. The presented results in Table 4 pertain to the inhibition zones of all control and active systems. Namely, the control coating Pull/CS and Pull/Gel layers (without the addition of hydrolats) have minimal (10.33 mm) or no inhibition zones of bacterial growth, respectively. The presence of a minimal suppressing effect on *S. aureus* cells of the first-mentioned layers can be attributed to chitosan, since this layer component has been investigated as a potential source of antimicrobials against *Staphylococcus* species [34,35]. As presented in Table 4, active Pull/CS and Pull/Gel coating layers (with the addition of hydrolats) exhibited significant inhibition zones (above the critical point of 26 mm), suggesting that the coating active layers have prodigious antimicrobial potential against *S. aureus*. In comparison with each other, the wider inhibition zone was observed for the active layer with chitosan, which can be a consequence of the mentioned growth-suppressing effect of the mentioned constituent (34.33 mm). These results are confirmation of the previously stated fact that selected hydrolats can be used as a source of antimicrobials for food-related systems such as formed coatings.

### 3.7. Antimicrobial Effect of an Active Coating System for Cheese Preservation

**Figure 7:**
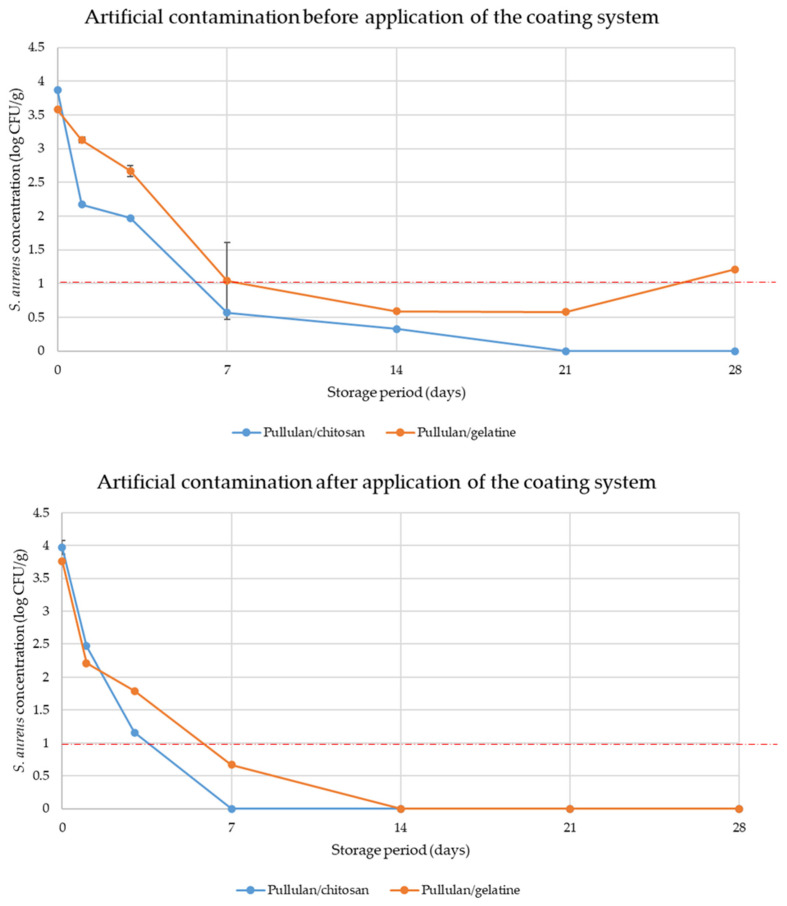
Performance evaluation of coating systems for controlling *Staphylococcus aureus* growth in cheese (all tests were conducted in triplicate; the error bars implicate standard deviations); dashed line (1 log CFU/g) is the maximum allowable limit of *S. aureus* in this context.

Since the antimicrobial potential of active Pull/CS and Pull/Gel coating layers was demonstrated (Table 4 and Figure 6), a coating procedure was applied for cheese samples (Figure 2). The results of the testing of coating efficiency are presented in Figure 7. Observing bacterial behavior in coating systems for both cases of artificial contamination (before and after application of coatings, respectively), a decreasing trend in bacterial concentration during the 28 days of the storage period in both cases was evident. Moreover, the impact of antimicrobial active coatings against *S. aureus* cells (before and after the application of coating systems) was proven.

### 3.8. Antimicrobial Effect of Edible Coatings as an Antimicrobial System for Cheese Samples

For the evaluation of the antimicrobial effect of formed coating systems, cheese samples were used, while artificial contamination was conducted with *Staphylococcus aureus*, one of the main foodborne bacteria. The chosen food product is highly susceptible to microbiological spoilage during storage, while the tested bacterium was selected for several reasons. Namely, the main concerns regarding *S. aureus* in targeted food samples arise when contaminated food workers or processing equipment come into contact with food during production or handling (contamination during milk collection, improper handling, unhygienic processing equipment, or post-pasteurization contamination, etc.). Inappropriate hygiene practices during the production process can introduce *Staphylococcus aureus* into the product and decrease the quality and shelf life of cheese [43]. This bacterium is commonly associated with cheese samples and is considered one of the potential contaminants in the cheese production process [48]. Cheese samples can provide an ideal environment for the growth of *Staphylococcus aureus*, especially certain types of cheese that have high moisture content and are stored at temperatures favorable for bacterial proliferation. Moreover, the intrinsic properties of cheese, such as its pH and water activity, can promote the survival and multiplication of *Staphylococcus aureus* during cheese ripening and storage [49]. Its presence in food can pose health risks to consumers since this bacterium produces heat-stable enterotoxins, which can lead to foodborne illnesses when ingested in sufficient quantities [50]. 

Therefore, coating systems (Pull/Chi and Pull/Gel) were tested in two different situations. The first situation involved artificial contamination before the application of the coating system, and the second after the application of the coating system. In this way, all real situations are covered: contamination during production, distribution, and storage. Both coating systems effectively limit bacterial growth, but some differences in inhibition pathways can be observed. The results presented here include the bacterial concentrations (in log CFU/g) for two types of coatings, Pull/CS and Pull/Gel, under the same storage conditions (Figure 7). 

In the case of artificial contamination before applying the coating system, both coatings contained similar bacterial concentrations, 3.87 log CFU/g and 3.58 log CFU/g for Pull/CS and Pull/Gel, respectively, at the beginning of the storage period (day 0). As the storage period progressed, both coatings demonstrated a decline in bacterial concentrations, indicating their antimicrobial effectiveness. For the Pull/CS coating, the bacterial concentration decreased steadily, reaching 0.33 log CFU/g on day 14 and complete elimination (0 log CFU/g) on day 21 and beyond. Similarly, the Pull/Gel coating exhibited a significant reduction in bacterial concentration, reaching 0.59 log CFU/g on day 14, followed by a slight increase in bacterial cells on day 21 and beyond. This demonstrates that both coatings possess effective antimicrobial properties, but formulation with chitosan has a biocide effect, while other tested formulations which included gelatine have only a bacteriostatic effect in the long-term setup.

Even when artificial contamination was conducted after the coating system was applied, both coatings demonstrated potent antimicrobial properties. For the Pull/CS coating, the bacterial concentration decreased to 1.16 log CFU/g on day 3, reaching complete elimination (0 log CFU/g) on day 7 and beyond. Similarly, the Pull/Gel coating exhibited a reduction in bacterial concentration to 1.79 log CFU/g on day 3, reaching complete elimination (0 log CFU/g) on day 14 and beyond. The results indicate that both Pull/CS and Pull/Gel coatings are effective in inhibiting bacterial growth regardless of the moment of contamination of the sample; the presented Pull/CS coating showed slightly better antimicrobial activity, achieving complete elimination of bacteria earlier compared with the Pull/Gel coating system. As expected, when contamination occurs after the application of an antimicrobial coating, the inhibitory effect is almost immediate. In this way, the chance of proliferation of bacteria that come into contact with the cheese during distribution and storage is significantly reduced. According to the obtained results, the strong antimicrobial potential of both pullulan/chitosan and pullulan/gelatine coatings was demonstrated, effectively inhibiting the growth of *S. aureus* over time and under artificial contamination conditions. These findings suggest potential applications for these coatings in various phases of the food industry process where bacterial control is essential. In both contamination scenarios, the coating systems consistently effectively limited bacterial proliferation, indicating the antimicrobial effect of the hydrolat mixture in the coating layers. By inducing a substantial reduction in bacterial concentrations over time, the shelf life of the cheese samples is prolonged and better microbial safety is ensured. It can be concluded that active substances present in plant hydrolats play a critical role in achieving these positive effects. Therefore, a complete antimicrobial assessment needs to be conducted before the selection of the most potent. 

## 4. Conclusions

This study presents advancements in the development of an active bilayer coating system, which represents a realistic approach to improving the microbial safety and shelf life of cheese products at the industrial level. A bilayer coating system has been formed to overcome the shortcomings of monolayer coatings based on the same biopolymers. The results of mechanical and barrier property characterization have shown that Pull/CS and Pull/Gel bilayer films possess improved values of tensile properties as well as lower permeability for water vapor in comparison to the films based on neat biopolymers. The mechanical, barrier, and thermal properties of prepared bilayer coatings are very similar. The improvement in mechanical and barrier properties of biopolymer films through the design of bilayer films confirms a hypothesis related to enhancing the functionality of these materials. The incorporation of lemongrass and curry plant hydrolats demonstrates a promising synergistic effect, effectively inhibiting the bacterial growth of the targeted bacterium *Staphylococcus aureus*. The antimicrobial assessment of the Pull/CS and Pull/Gel coatings, both under artificial *S. aureus* contamination before and after application, exposes their potent efficacy in inhibiting bacterial growth over time. While both coatings demonstrate effective antimicrobial properties, the Pull/CS coating exhibits a biocidal effect, achieving complete elimination of the bacterium earlier than the Pull/Gel coating, which shows a bacteriostatic effect in the long-term setup. These results emphasize the significance of considering the specific antimicrobial properties of each formulation, with the Pull/CS coating demonstrating slightly superior activity. Overall, the incorporation of hydrolat mixtures in the coating layers consistently limits bacterial proliferation, extending the shelf life of cheese samples and enhancing microbial safety in various phases of the food industry process. Although the rapid cell inactivation of this bacterial indicator of hygiene during cheese processing demonstrates the effectiveness of the coating system, further experiments are warranted to assess its broader spectrum against potential contaminants in cheese, especially fungi. Improved barrier, mechanical, and antimicrobial properties of investigated coatings resulted in an extended shelf life of cheese and enhanced safety of sliced cheese for consumers. However, the influence of hydrolat application in the coating system on the sensory properties of cheese has to be investigated in the following stage of this investigation. This study provides a platform for further industrial application, presenting economic and logistical advantages by mitigating product waste and elevating quality over an extended duration.

## Figures and Tables

**Table 1:** Tensile strength and elongation at break results.

Samples	Tensile Strength (N/mm^2^)	Elongation at Break (%)
Pull	4.83 ± 2.7	38.12 ± 4.1
CS	7.23 ± 2.4	36.72 ± 3.4
Gel	8.35 ± 3.2	32.65 ± 2.9
Pull/CS	31.20 ± 3.6	11.23 ± 3.5
Pull/Gel	41.67 ± 3.3	10. 55 ± 4.2

**Table 2:** Thickness, WVP, and MS values for monolayer and bilayer films.

Samples	Thickness (mm)	WVT (g/h·m·Pa)	Moisture Content (%)
Pull	0.34 ± 0.01	1.47 × 10^−3^ ± 0.17	2.8 ± 0.07
CS	0.36 ± 0.02	1.35 × 10^−3^ ± 0.25	3.6 ± 0.06
Gel	0.37 ± 0.01	1.5 × 10^−3^ ± 0.15	3.1 ± 0.07
Pull/CS	0.38 ± 0.02	2.42 × 10^−4^ ± 0.21	4.8 ± 0.09
Pull/Gel	0.38 ± 0.02	2.11 × 10^−4^ ± 0.17	3.9 ± 0.09

**Table 3:** DTG peak maxima values for monolayer and bilayer films.

Samples	T_dmaxI_ (°C)	T_dmaxII_ (°C)	T_dmaxIII_ (°C)	Residual Mass
Pull	107	271	539	0.02
CS	109	274	541	0.02
Gel	111	269	544	0.02
Pull/CS	119	311	545	0.02
Pull/Gel	118	309	542	0.02

**Table 4:** Disk diffusion test of plant-based hydrolats and coating systems against *Staphylococcus aureus* (all tests were conducted in triplicate).

Sample		Inhibition Zone (mm) *
Hydrolate	Lemongrass	34.33 ± 0.56
	Curry plant	25.00 ± 1.00
Control coating layer (without the addition of hydrolates)	Pull/CS	10.33 ± 1.00
	Pull/Gel	nd **
Active coating layer (with the addition of hydrolates)	Pull/CS	31.00 ± 1.00
	Pull/Gel	27.33 ± 0.56

* Results are presented as average values ± standard deviations; ** nd—not detected.

## Data Availability

Data will be made available on request.

## Supplementary Materials

The following supporting information can be downloaded at: https://www.mdpi.com/article/10.3390/polym16020178/s1.
