# Supplementary material for: Comparison of the Properties of Pullulan-Based Active Edible Coatings Implemented for Improving Sliced Cheese Shelf Life
Source: Polymers (Basel). 2024 Jan 7;16(2):178. doi: 10.3390/polym16020178 (PMC10821112; doi:10.3390/polym16020178)
Supplement: Supplementary file 1 [file polymers-16-00178-s001.zip › polymers-2741542-supplementary.pdf]

File :C:\MSD DATA\2022\Milica\Maj\Cymbo pogoncitatus 2021 H LN.D
Operator :
Acquired : 31 May 2022 10:55 using AcqMethod ADAMS 50.M
Instrument : GCMS
Sample Name: CYMBO POGONCITATUS 2021
Misc Info :
Vial Number: 55

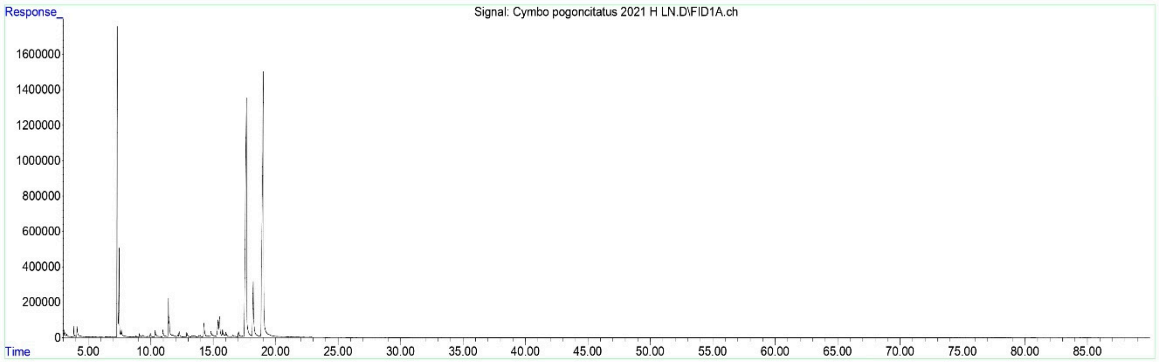

Figure S1. Chromatogram of lemongrass hydrolat.

File :C:\MSD DATA\2023\Milica\Hidrolati 2022\H Helicrysum italicum
... 2021 50.D
Operator :
Instrument : GCMS
Acquired : 3 Feb 2023 16:48 using AcqMethod ADAMS 50.M
Sample Name: H Helicrysum italicum 2021
Misc Info :

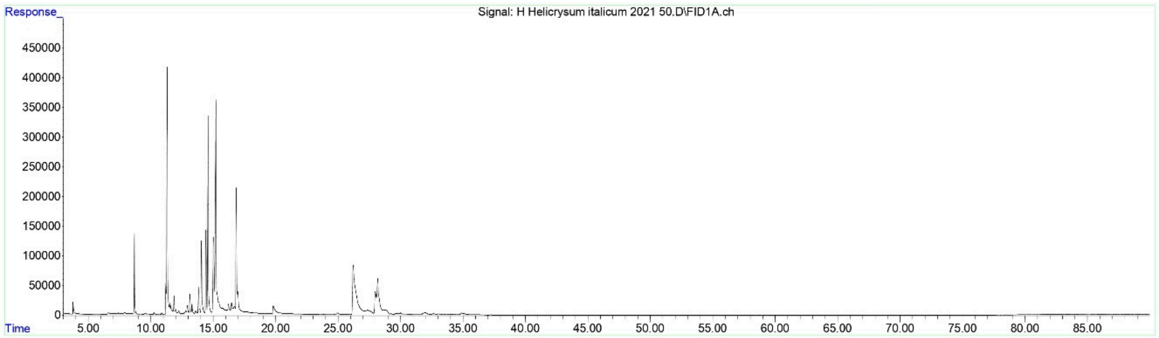

Figure S2. Chromatogram of curry plant hydrolat.
